# Aneurysms of splenic artery in a patient with autosomal dominant polycystic kidney disease

**DOI:** 10.1007/s40620-024-01946-3

**Published:** 2024-05-08

**Authors:** Julia Borowiecka, Zofia Jankowska, Monika Gradzik, Mariusz Niemczyk

**Affiliations:** 1 Department of Transplantology, Immunology, Nephrology, and Internal Medicine, Medical University of Warsaw, Warsaw, Poland; 2 Department of Clinical Radiology, Medical University of Warsaw, Warsaw, Poland

A 37-year-old woman with autosomal dominant polycystic kidney disease (ADPKD) and normal kidney function, with well-controlled arterial hypertension, and rupture of an intracranial aneurysm at 27 years of age presented to our institute. Ultrasound led to a suspicion of two splenic artery aneurysms, which was subsequently confirmed by computed tomography (Fig. 1). The patient was referred to a vascular surgeon who recommended observation alone. To date, the aneurysms remain clinically silent.

**Fig. 1:**
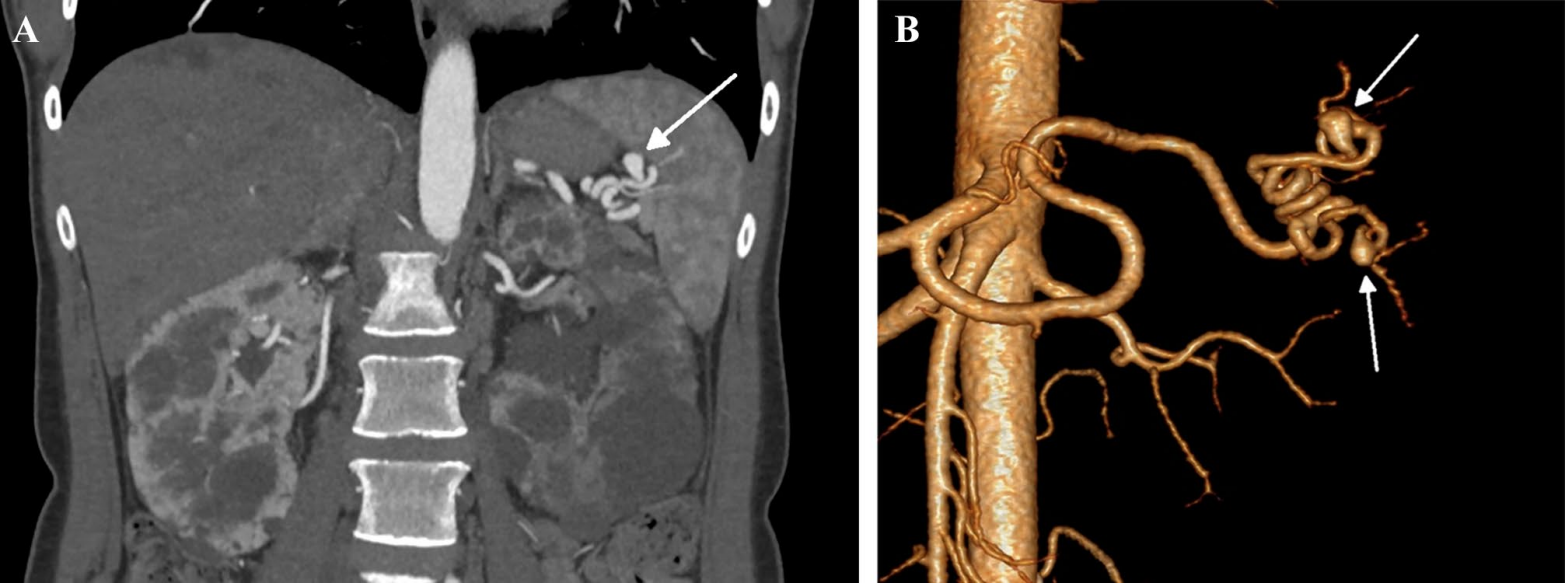
**A** Contrast-enhanced computed tomography of the abdominal cavity, coronal plane. Aneurysm of the splenic artery (arrow). Polycystic kidneys. **B** Contrast-enhanced computed tomography (3D reconstruction). Two aneurysms of the spenic artery (arrows)

ADPKD is the most common monogenic disease of the kidney. It affects approximately 1 in every 1000 people. As well as being a cause of chronic kidney disease, it is accompanied by numerous extra-renal manifestations, including aneurysms. Due to possible dramatic consequences of rupture, intracranial aneurysms, observed in approximately 10% of ADPKD patients [1], attract the most attention. However, aneurysms can also be found in other arteries [2].

Knowledge of the pathogenesis of aneurysms in ADPKD remains piecemeal. ADPKD is caused by a mutation in the PKD1 or PKD2 gene, leading to disturbed structure and function of their protein products, polycystin-1 (PC-1), or polycystin-2 (PC-2), respectively. Mutations in PKD1 or PKD2 lead to dysregulation of various intracellular signaling pathways. Both PKD1 and PKD2 are expressed in the smooth muscle and myofibroblasts of blood vessels, and polycystins play an important role in maintaining the normal structure and function of blood vessels. Additionally, involvement of vascular enothelial growth factor (VEGF), collagen genes and genes of the TGF-β pathway in vascular remodeling was shown; however, exact mechanisms were not elucidated. These changes, together with arterial hypertension, which is common in ADPKD, may result in aneurysm development [S1, S2—see Suppl. file].

Rupture of an intracranial aneurysm in a patient’s medical history is considered a risk factor for the development of new intracranial aneurysms in ADPKD [1]. Our case reminds us that new aneurysms should also be expected in other locations.

## Supplementary Information

Below is the link to the electronic supplementary material.

Supplementary file1 (DOCX 14 KB)

## Data Availability

Additional data are available from the corresponding author on justifiable request.
