# Supplementary material for: Aneurysms of splenic artery in a patient with autosomal dominant polycystic kidney disease
Source: J Nephrol. 2024 May 8;37(8):2355–6. doi: 10.1007/s40620-024-01946-3 (PMC11649809; doi:10.1007/s40620-024-01946-3)
Supplement: Supplementary file 1 — Supplementary file1 (DOCX 14 KB) [file 40620_2024_1946_MOESM1_ESM.docx]

SUPPLEMENTARY FILE - REFERENCES

S1. Samuel N, Radovanovic I. Genetic basis of intracranial aneurysm formation and rupture: clinical implications in the postgenomic era. Neurosurg Focus. 2019;47(1):E10.

S2. Zhu J, Liu F, Mao J. Clinical findings, underlying pathogenetic processes and treatment of vascular dusfunction in autosomal dominant polycystic kidney disease. Ren Fail. 2023;45(2):2282027.
